# Self-medication for migraine: A nationwide cross-sectional study in Italy

**DOI:** 10.1371/journal.pone.0211191

**Published:** 2019-01-23

**Authors:** Paola Brusa, Gianni Allais, Cecilia Scarinzi, Francesca Baratta, Marco Parente, Sara Rolando, Roberto Gnavi, Teresa Spadea, Giuseppe Costa, Chiara Benedetto, Massimo Mana, Mario Giaccone, Andrea Mandelli, Gian Camillo Manzoni, Gennaro Bussone

**Affiliations:** 1 Department of Science and Technology of Drugs, University of Turin, Turin, Italy; 2 Order of Pharmacists of Turin, Turin, Italy; 3 Department of Surgical Sciences, Women’s Headache Center, University of Turin, Turin, Italy; 4 FI.CEF Onlus, Italian Headache Foundation, Milan, Italy; 5 Epidemiology Unit, ASL TO3, Grugliasco (Turin), Italy; 6 ATF Informatics, Cuneo, Italy; 7 FOFI, Federation of the Orders of Italian Pharmacists, Rome, Italy; Universiti Sains Malaysia, MALAYSIA

## Abstract

Headache disorders are considered the second leading cause of years lived with disability worldwide, and 90% of people have a headache episode at least once a year, thus representing a relevant public health priority. As the pharmacist is often the first and only point of reference for people complaining of headache, we carried out a survey in a nationwide sample of Italian pharmacies, in order to describe the distribution of migraine or non-migraine type headaches and medicines overuse among people entering pharmacies seeking for self-medication; and to evaluate the association, in particular of migraine, with socio-demographic and clinical characteristics, and with the pathway of care followed by the patients. A 14-item questionnaire, including socio-demographic and clinical factors, was administered by trained pharmacists to subjects who entered a pharmacy requesting self-medication for a headache attack. The ID Migraine^™^ Screener was used to classify headache sufferers in four classes. From June 2016 to January 2017, 4424 people have been interviewed. The prevalence of definite migraines was 40%, significantly higher among women and less educated people. About half of all headache sufferers and a third of migraineurs do not consider their condition as a disease and are not cared by any doctor. Among people seeking self-medication in pharmacies for acute headache attacks, the rate of definite or probable migraine is high, and a large percentage of them is not correctly diagnosed and treated. The pharmacy can be a valuable observatory for the study of headaches, and the first important step to improve the quality of care delivered to these patients.

## Introduction

Headache disorders are considered by The Global Burden of Disease study as the second leading cause of years lived with disability worldwide [1]. The World Health Organization declared that headache disorders are ubiquitous, prevalent and disabling but despite this they are under-recognized, under-diagnosed and under-treated all over the world [2]. Ninety percent of people have a headache episode at least once a year and 40% are serious or disabling headaches. Headaches cause a loss of productivity at work, a reduction in social interactions and a deterioration in the quality of life [2]. Globally, it has a prevalence of 47% and is the most common neurological syndrome for which people turn to their family doctor while only 16% consult a neurologist [2]. Recent epidemiological studies have shown that the phenomenon of migraine may be compared to an iceberg: the top represents correctly diagnosed cases while the submerged part is made up of those subjects that have never consulted a doctor or that, having done so, have not received a correct diagnosis [3, 4]. Furthermore, in Italy, many migraineurs make a wrong self-diagnosis assuming cervical spine pathology as the cause [5]. This explains why the majority of migraineurs make use of self-medication, taking over the counter (OTC) analgesics to treat their headache [6,7]. The lack of a proper treatment for headache, however, can lead to an overuse of acute pain-relief medicine and to the medication overuse headache (MOH), an even more disabling chronic disorder [8,9].

In Europe, the total annual cost of headache amongst adults aged 18–65 years was calculated, in a study of 2012, at €173 billion [10]. In Italy, among the neurological disorders, migraine ranked first for direct and indirect costs with a total expenditure amounting to € 3.5 billion per year [11]. An Italian study conducted on 135 patients with MOH, demonstrated that the direct costs, calculated by medications for acute treatment and prophylaxis, diagnostic procedures, visits, complementary treatments, informal care, were around 415 €/month per person; indirect costs were 530 €/month, and were mostly due to presenteeism (350 €, 66.3%), i.e. days worked with headache, rather than to absenteeism, lost workdays (160 €, 33.7%) [12]. Thus, headaches in general and migraines in particular represent not only an enormous limitation for the affected person, but a problem for the entire community [13].

The pharmacist is often the first and only point of reference for the patient complaining of headache, and participates in the management of this widespread disorder. He/she should be able to understand the problem raised by the patient, recommend the most appropriate symptomatic treatment and, if necessary, refer the patient to a general practitioner or a specialist. With proper training, the community pharmacist could be responsible for identifying patients suffering from suspected migraine symptoms that have not been diagnosed, owing to poor awareness about the problem and lack of specialist advice [14]. Community pharmacies are also the ideal site where the risks associated with inappropriate self-medication could be prevented. Community pharmacists are strategically positioned as they have a clear overview of the medications taken by a patient, whether prescribed by a physician or purchased directly [15].

Considering the potential role of community pharmacists, and his/her strategical role as first contact with headache sufferers, a preliminary study was carried out in Piedmont in 2014, with the objective of analyzing the patterns of use of drugs in subjects who entered a pharmacy requesting OTC medications in order to treat a headache attack [16]. Thanks to the findings of that study, both from an epidemiological perspective and a public health policy point of view [16, 17], the decision was taken to expand the scale of the model to a national level. The detailed protocol of the national survey has already been reported [18]. The objectives of the present paper are to describe the distribution of migraine or non-migraine type headaches and medicines overuse among people entering pharmacies seeking for self-medication; and to evaluate the association, in particular of migraine, with socio-demographic and clinical characteristics, and with the pathway of care followed by the patients.

## Materials and methods

**Fig 1:**
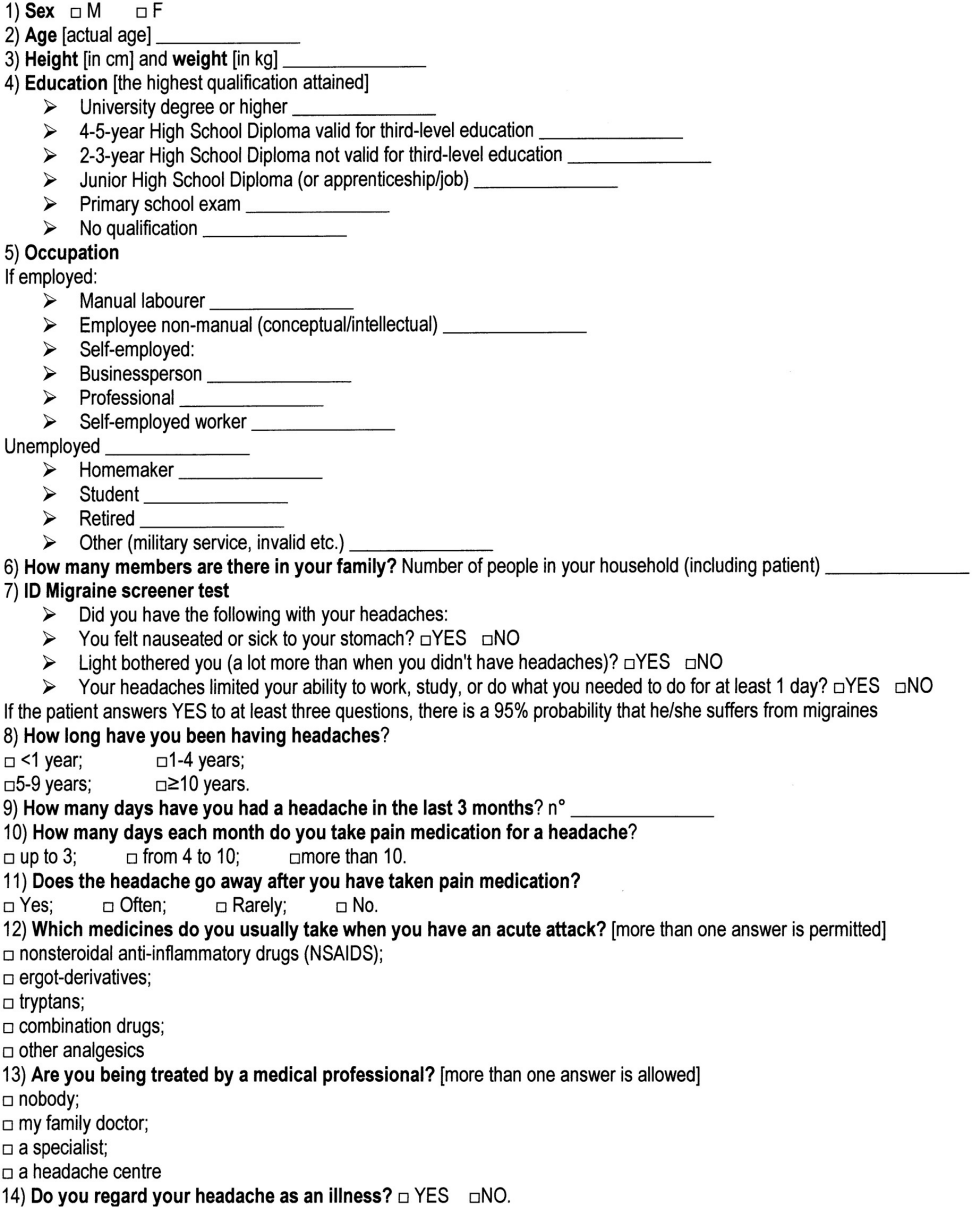
Questionnaire.

The study was designed as a cross-sectional survey based on face-to-face interviews using questionnaires drawn up by experts and based on the scientific literature. Briefly, the questionnaire, reported in Fig 1, covered the following points: the socio-economic situation of the recruited subjects; the ID Migraine screener test (ID-MS) [19,20]; the frequency of attacks, the drugs usually taken and the healthcare professional, if any, responsible for the management of the subject’s headache.

Given the nature of the centres responsible for recruiting the subjects (community pharmacies), a single national online training course was employed to prepare the pharmacists in order to ensure that the data were collected as homogeneously as possible. At the end of the training course, each pharmacist had to pass a final test before being enrolled in the project. Pharmacists were selected on a voluntary basis, and did not receive any payment for their participation in the survey.

The questionnaire, was administered to subjects who entered a pharmacy requesting self-medication for a headache attack, from June 2016 to January 2017. The subjects were recruited at a maximum rate of five patients per pharmacy per month. [18]

Age at enrolment was categorized in three levels: less than 30 years, 30–54 years and more than 54 years. Educational level was measured by means of the maximum attained classification and classified according to the International standard classification [21] in three levels: low, including people up to the completion of lower secondary school (ISCED 0–2); medium, including higher secondary school diplomas (ISCED 3); and high, with any university degree (ISCED 5–6).

The clinical section included information about symptoms, occurrence and severity of headaches, together with the items of the ID-MS, resulting in a four-level variable that classifies patients as suffering from definite migraine, probable migraine, unlikely migraine, or other headaches [19, 20]. The variable “Number of days with headache in the last three months” was categorized in two ways:

1. 0–44 days and ≥45 days in accordance with the criteria laid down by the International Headache Society (IHS) classification [22] to distinguish episodic migraine (EM) (<15 days per month) from chronic migraine (CM) (≥15 days per month);
2. <11days, 11–29 days, 30–60 days e >60 days in line with the time limits recently recommended by Manzoni et al [23] in order to distinguish respectively: infrequent EM (≤3days per month), frequent EM (>3 but <10 days per month), very frequent migraine—or CM- (≥10 but ≤20 days per month) and transformed migraine (TM) (>20 days per month).

People who declared to take pain medications for more than 10 days a month were classified as medicines overusers (MOs).

Finally, as for pathway of care, patients were asked which medicines were usually taken in case of an acute attack and whether they were monitored by a professional for the treatment of headache. More details on the protocol of the survey are reported elsewhere [18].

To describe the association of age, pathway of care and clinical information with the type of headache, we computed conditional relative frequencies and related chi-square p-values, stratified by gender. The Monte-Carlo exact test was applied if the cells with expected conjoint frequencies lower than 5 were more than 20%. Statistical analyses were carried out using the SAS software (version 9.2).

### Patient involvement

The study was designed in order to describe the distribution of migraine or non-migraine type headaches and medicines overuse among people seeking for self-medication, and to evaluate its association with individual characteristics and with the pathway of care followed by the patients. The study was approved by the national association of cephalalgic patients. The study was also approved and then financed by the Federation of the Orders of Italian Pharmacists (FOFI). Approval by an ethics committee was not required because the subjects participated in the study on a voluntary basis and they were informed on the characteristics and the purpose of the study. The questionnaire was anonymous, personal data were not collected and there is no way to trace back the answers to a specific responder.

## Results

The development of the study at a national level expanded the number of participating pharmacists to 610, operating in 514 community pharmacies situated in 19 out of the 20 Italian regions. Out of 610 trained pharmacists, 445 collected at least one questionnaire and participated in this survey; overall, 4424 questionnaires had been correctly completed.

The prevalence of migraine and headache according to the ID-MS classification, by socio-demographic characteristics is summarized in Table 1. The study population included mostly women (74%), highly educated people (only 25% in the lowest level), residing in the North of Italy (84%), and with a mean age of 45 years. Among people asking for self-medication because of headache, 40% has a definite migraine and 31% a probable migraine, while the remaining 30% is unlikely to have a migraine or has other forms of headaches.

**Table 1:** Prevalence (%) of migraine according to the ID-MS classification by socio-demographic characteristics.

Socio-demographic characteristics	migraine classes according to the ID-MS classification				Total		p-value §
	other headaches (N = 389)	unlikely migraine (N = 898)	probable migraine (N = 1372)	definite migraine (N = 1765)			
	**%**	**%**	**%**	**%**	N	%	
**Gender**							
Female	7.0	18.1	29.9	45.0	3265	73.8	<0.0001*
Male	13.7	26.4	34.3	25.6	1159	26.2	
**Educational level**							
Low	8.2	20.1	28.3	43.4	1112	25.1	0,0001*
Medium	8.9	18.7	30.7	41.7	1900	42.9	
High	9.1	22.7	33.5	34.7	1412	31.9	
**Residence area**							
North	9.3	19.6	31.3	39.8	3726	84.2	0,004*
Center, South and Isles	6.2	24.1	29.4	40.4	698	15.8	
**Age in years**					mean value		
mean	46.0	45.5	44.6	45.1	45.1		0.21
Total (%)	8.8	20.3	31.0	39.9	4424		

^§^ p-values referred to the chi-square or Monte-Carlo exact tests on the distribution of the socio-demographic characteristics across all ID-MS classes

* Statistically significant

The ID-MS shows a strong association with gender: almost half of the women (45%) were classified as definite migraine vs. only a quarter of men (26%), while the percentage of men with other types of headaches was twice that of women (14% vs. 7%). The overall ratio of females to males equals 2.8:1, but it is 4.9:1 among subjects with definite migraine and 1.4:1 for non-migraine sufferers.

Within the sample, the rate of definite migraines was significantly higher among the two lower educational levels than among people with university qualifications (44% and 42% vs. 35%); there were slightly more other headaches in people living in the North compared to the rest of Italy (9% vs. 6%). Mean age was homogeneously distributed across the classes of migraine according to the ID-MS classification.

When looking at men and women separately (Table 2), a chronic headache (at least 45 headache days over the previous three months) is reported in 5.4% of men (n = 63) and in 7.9% of women (n = 257) and MOs in 14.9% (n = 173) and 18.8% (n = 613), respectively. Less than 10% (130 men and 189 women) have had this condition for less than one year, while about 60% have been affected for at least 10 years (563 men and 2039 women).

**Table 2:** Prevalence (%) of migraine according to the ID-MS classification by gender and clinical characteristics.

Clinical characteristics	MALE						FEMALE					
		migraine classes						migraine classes				
		other headaches N = 159	unlikely migraine N = 306	probable migraine N = 397	definite migraine N = 297			other headaches N = 230	unlikely migraine N = 592	probable migraine N = 975	definite migraine N = 1468	
	N	%	%	%	%	*p-value* §	N	%	%	%	%	*p-value* §
**Age**												
≤29	138	11,6	27,5	34,1	26,8	0,67	447	6,7	18,3	33,3	41,6	0,16
30–54	724	13,8	25,7	33,4	27,1		2092	6,5	18,0	29,3	46,2	
≥55	297	14,5	27,6	36,4	21,5		726	9,0	18,3	29,3	43,4	
**Number of years with headache**												
<1	130	36,9	31,5	22,3	9,2	<0.0001*	189	20,6	37,0	26,5	15,9	<0.0001*
1–4	268	14,6	30,6	34,3	20,5		537	10,2	25,0	32,6	32,2	
5–9	198	11,1	22,7	38,9	27,3		500	6,8	16,8	33,8	42,6	
≥10	563	8,9	24,5	35,3	31,3		2039	5,0	14,9	28,5	51,6	
**Number of days with headache in the last three months**												
<45	1096	14,2	26,7	34,3	24,7	0,01*	3008	7,3	18,6	30,3	43,9	0,0002*
≥45	63	4,8	20,6	33,3	41,3		257	3,9	13,2	25,3	57,6	
**Number of days with headache in the last three months**												
<11	871	15,8	28,6	33,6	21,9	<0.0001*	2153	9,0	20,9	30,5	39,6	<0.0001*
11–29	177	6,8	22,0	35,6	35,6		638	2,8	12,9	29,2	55,2	
30–60	74	9,5	13,5	40,5	36,5		333	4,5	11,1	30,0	54,4	
>60	37	5,4	21,6	29,7	43,2		141	2,8	15,6	23,4	58,2	
**Number of types of medicines**												
1	935	15,1	28,2	33,9	22,8	<0.0001*	2531	8,0	20,3	30,9	40,8	<0.0001*
2	197	8,6	18,8	35,5	37,1		620	4,5	11,1	27,9	56,5	
≥3	27	3,7	18,6	37,0	40,7		114	.0.0	7,9	17,5	74,6	
**Medication Overuse Headache**												
No	986	15,0	27,9	34,2	22,9	<0.0001*	2652	8,1	20,0	30,8	41,0	<0.0001*
Yes	173	6,4	17,9	34,7	41,0		613	2,4	10,0	25,6	62,0	
**Type of drug**												
NSAIDs	272	9,9	20,6	31,6	37,9	<0.0001*	964	3,4	12,6	25,1	58,9	<0.0001*
Ergot-derivatives	28	7,1	28,6	28,6	35,7	0,5	123	1,6	8,1	23,6	66,7	<0.0001*
Triptans	220	4,1	15,0	33,6	47,3	<0.0001*	855	1,5	7,0	24,9	66,5	<0.0001*
Combination drugs	82	9,8	20,7	36,6	32,9	0,26	284	2,5	14,1	20,8	62,7	<0.0001*
Other analgesics	197	13,7	22,8	34,0	29,4	0,48	564	6,9	17,6	27,3	48,2	0,35
**Pain medication solved the problem**												
Yes	613	18,9	32,8	32,0	16,3	<0.0001*	1384	11,8	25,1	32,6	30,6	<0.0001*
Often	360	10,0	19,7	39,4	30,8		1055	3,7	16,0	29,8	50,5	
Rarely	128	5,5	16,4	29,7	48,4		538	3,7	8,0	25,8	62,5	
No	58	0,0	22,4	36,2	41,4		288	2,8	11,5	24,7	61,1	
**The headache is a disease**												
No	677	20,4	33,1	34,4	12,1	<0.0001*	1545	12,4	26,9	33,7	27,0	<0.0001*
Yes	482	4,4	17,0	34,0	44,6		1720	2,2	10,3	26,4	61,1	
**Who takes care of the patient**												
Nobody	676	17,9	32,7	32,5	16,9	<0.0001*	1547	10,9	26,2	32,6	30,3	<0.0001*
General practitioner	318	8,2	18,6	39,3	34,0	<0.0001*	1030	4,2	12,1	27,6	56,1	<0.0001*
Specialist	135	8,1	12,6	35,6	43,7	<0.0001*	533	2,8	10,1	25,9	61,2	<0.0001*
Headache Center	82	3,7	20,7	32,9	42,7	0,0005*	365	2,7	5,5	24,1	67,7	<0.0001*

^§^ p-values referred to the chi-square or Monte-Carlo exact tests on the distribution of the clinical characteristics across all ID-MS classes

* Statistically significant

Almost all the covariates are significantly associated with the type of headache, except for age and some types of drugs (as ergot-derivatives, combination drugs and other analgesics). In particular, a higher duration and frequency of headache (higher number of years and of days in each month) is associated with higher prevalence of “definite migraine”, as well as a higher number of drugs. These associations are particularly evident among women. Also MOs were significantly overrepresented among subjects with definite or probable migraine (76% among men and 88% among women); only 6.4% of men MOs and 2.4% of women suffered from non-migraine headaches.

As for therapies, triptans and nonsteroidal anti-inflammatory drug (NSAID) users are mostly affected by more severe migraines (81% and 70%, respectively among men; 91% and 84% among women), but only 38% of subjects with definite migraine take triptans in order to block the attack. On the other hand, men use all the other types of drugs independently of the migraine class, while women with other forms of headaches tend to use only other analgesics. Headaches classified as probable or definite migraines are more often not resolved by pain medication, other types of headaches are usually blocked with symptomatic medications.

Finally, about 50% of all patients and 30% of those who suffer from definite migraines do not believe that headache is a disease and do not require medical care, especially among men. Indeed, 49% of men and 63% of women who are not cared by any doctor are likely to be serious migraineurs. Of those who do consult a doctor, half goes to the general practitioner and the other half is in care with a specialist or at a headache centre. For both genders, the higher the ID-MS class, the higher the percentage of patients who believe that migraine is a disease and who are in care by either a physician or a Headache Center.

## Discussion

In accordance with other studies [13] we report that women are more likely than men to suffer from headaches, particularly from migraines. In both genders, there appears to be a trend towards mainly non-occasional headaches and migraines, existing for a long time and accompanied by an important prevalence of chronic headache. Since the epidemiological data reported in the literature indicate a prevalence of chronic migraine in the general population of only 2%, must be true that the migraineurs entering a pharmacy and enrolled in this survey have a relatively severe form of migraine.

Despite the specificity and usefulness of triptans, which have been available on the market for over 25 years, we found that only one third of severe migraineurs use this class of medication. This is in line with data from Fisher *et al*., where it has been reported that almost three quarters of patients admitted for a preliminary observation at the Headache Outpatient Clinic had never taken triptans before [24].

It has long been known that the overuse of symptomatic medications can worsen migraine, until it loses the typical episodic temporal pattern and transform it into a chronic headache [25]. In this context we can speak of MOs. In our study, approximately one quarter of subjects with definite migraine declared that they took symptomatic medications at a frequency deemed to be medication overuse by the criteria in the IHS classification [26]. If we consider the worsening of the migraine due to medication overuse and the difficulty in detecting migraineurs who often self-medicate without consulting a doctor, this high percentage of migraineurs MO entering a pharmacy is clear evidence of the fundamental importance of this site as the first point of contact for medicines overuse management.

About half the subjects included in our study believe that their headache is not a disease and manage their pain by themselves without consulting a medical professional. The migraineurs have a greater awareness of this condition as a disease, and are more likely to be treated by medical professionals, although a third of subjects with definite migraine does not receive any medical consultation, thus giving wide space to interventions aimed to improve the quality and appropriateness of care delivered to these patients. The fact that this condition mainly affects middle-aged subjects very busy from the working point of view and generally in good health except for migraine problems, reduces the chance of consulting a doctor. An interesting study conducted in the US in 1998 [27] indicated that a mere 50% of migraineurs in the general population were in care with a doctor. They found, moreover, that even amongst those who consulted a doctor, the majority either had been misdiagnosed, or had been diagnosed correctly but were being treated with an inappropriate therapy or, even having been diagnosed correctly and receiving a correct therapy, had not been going back for regular follow-up examinations. The study arrived at the bitter conclusion that a tiny percentage of migraineurs (6%) were managed appropriately. The higher percentage of migraineurs consulting a doctor found by our study is probably due to the fact that not the general population was analysed but subjects were entered into a pharmacy looking for pain relief and, therefore, affected by a more severe form of migraine.

To sum up, the results of our investigation highlight that females make up the great majority of subjects entering a pharmacy seeking pain relief; approximately 40% of all cases were subjects with definite migraine and for many of them the migraine symptoms had been present for many years and with frequent attacks, which did not always respond to symptomatic therapies. About half of headache sufferers and about a third of migraineurs do not consider their condition as a disease and tend to self-medicate, which leads to inappropriate treatment of the condition.

A limit of our study is that the participating pharmacists were enrolled into the study on a volunteer basis, and are not a representative sample of the Italian population of pharmacists, as the rate of participation was much higher in Northern regions of Italy compared to Central or Southern. Consequently, patients interviewed cannot be considered as representative of the Italian population seeking for self medication in a pharmacy. Unfortunately, we cannot estimate the magnitude of this source of bias, as there are no available data to compare the characteristics of population entering in pharmacy with the general population. On the other hand, the fact that all interviewing pharmacists were volunteer and underwent a formal training in interviewing headache suffering customers should ensure a high level of quality of data collected.

An original contribution of our work is that we showed the strength of the pharmacies as epidemiological sentinels, given their capillary network on the field, and their contact with a wide range of users. This allows any pharmacy-based study to develop a snapshot close to the real-life situation and far more accurate than that obtained from data based in specialist centres, enabling a choice of clinical strategies that are coherent with the real needs of the local population.

## Conclusion

The pharmacy can be not only a valuable observatory for the study of headaches, but can also represent, through the counselling that a formed pharmacist might give, the first important step in seeking to build a good management relationship for subjects with headache, especially so for undiagnosed migraineurs; their referral in the first instance to the general practitioner and then to specialists and headache centres, may prevent the onset of chronic migraine and medication overuse headache.

## Abbreviations

CM: chronic migraine
EM: episodic migraine
ID-MS: ID Migraine^™^ Screener
IHS: International Headache Society
MOH: medication overuse headache
MOs: medication overusers
NSAID: nonsteroidal anti-inflammatory drug
OTC: over the counter
TM: transformed migraine
